# National Environmental Public Health Tracking Program: Bridging the Information Gap

**DOI:** 10.1289/ehp.7144

**Published:** 2004-08-03

**Authors:** Michael A. McGeehin, Judith R. Qualters, Amanda Sue Niskar

**Affiliations:** Division of Environmental Hazards and Health Effects, National Center for Environmental Health/Agency for Toxic Substances and Disease Registry, Centers for Disease Control and Prevention, Atlanta, Georgia, USA

**Keywords:** environmental monitoring, environmental public health surveillance, information system integration, tracking

## Abstract

In January 2001 the Pew Environmental Health Commission called for the creation of a coordinated public health system to prevent disease in the United States by tracking and combating environmental health threats. In response, the Centers for Disease Control and Prevention initiated the Environmental Public Health Tracking (EPHT) Program to integrate three distinct components of hazard monitoring and exposure and health effects surveillance into a cohesive tracking network. Uniform and acceptable data standards, easily understood case definitions, and improved communication between health and environmental agencies are just a few of the challenges that must be addressed for this network to be effective. The nascent EPHT program is attempting to respond to these challenges by drawing on a wide range of expertise from federal agencies, state health and environmental agencies, nongovernmental organizations, and the program’s academic Centers of Excellence. In this mini-monograph, we present innovative strategies and methods that are being applied to the broad scope of important and complex environmental public health problems by developing EPHT programs. The data resulting from this program can be used to identify areas and populations most likely to be affected by environmental contamination and to provide important information on the health and environmental status of communities. EPHT will develop valuable data on possible associations between the environment and the risk of noninfectious health effects. These data can be used to reduce the burden of adverse health effects on the American public.

## Why We Need an Environmental Public Health Tracking Network

At the turn of the 20th century, the American population faced significant health challenges. The recent shift in population from rural to urban that accompanied industrialization resulted in overcrowding in dilapidated housing served by inadequate water supplies and nonexistent waste disposal systems. These conditions led to continued outbreaks of infectious diseases that ravaged the population. In 1900 one-third of all deaths were caused by pneumonia, tuberculosis, or diarrhea, and 40% of these deaths were among children younger than 5 years (Bureau of the Census 1906). After the discovery of the “germ theory” of disease, much of the dramatic decrease in mortality from infectious disease in Western civilization was attributable to environmental public health measures such as disinfection of water, food safety regulations, and housing improvements, among others [Centers for Disease Control and Prevention (CDC) 1999].

The last half century witnessed a dramatic shift in the health burden of the U.S. population from infectious diseases to diseases such as cancer, birth defects, and asthma, many of which may be associated with environmental exposures. During the same period, advances in industrial science and technology led to the development and production of tens of thousands of chemical compounds. Unheard of 50 years ago, these chemicals are now ubiquitous in our air, water, food, workplaces, and homes. Mankind has benefited substantially from these products, but the health implications of long-term exposure to low levels of these compounds are not well understood. The American people feel strongly that the environment plays a role in their health. A poll taken in 1999 by the Pew Charitable Trusts found that 87% of Americans believed that environmental factors such as pollution cause increased rates of diseases and health problems (Pew Charitable Trusts 1999).

In September 2000, after 18 months of review, the Pew Environmental Health Commission released a report on the state of environmental public health in the United States (Environmental Health Tracking Project Team 2000). The commission found that the environmental public health system was fragmented, neglected, and ineffective. The report stated that the current system does not have the capability to respond adequately to environmental threats. The first of a number of recommendations made by the commission called on the federal government to establish a national environmental public health tracking (EPHT) network to link information on environmentally related diseases, human exposures, and environmental hazards. The information from this tracking network would be used to respond to, and eventually reduce, the burden of these diseases on the nation’s population. The commission estimated the cost of this tracking network to be $275 million annually.

Public health surveillance or tracking systems are critical in preventing and controlling disease in populations. Accurate and timely surveillance data permit public health authorities to determine disease impacts and trends, recognize clusters and outbreaks, identify populations and geographic areas most affected, and assess the effectiveness of public health interventions (Teutsch 2000). Most of the public health surveillance currently in place in the United States focuses on infectious diseases. We urgently need a more comprehensive national approach to the collection and analysis of noninfectious disease data and the integration of that information with environmental hazard monitoring and exposure data. The availability of these types of data in a standardized tracking network will enable researchers and health authorities to begin to understand the possible associations between the environment and adverse health effects.

## An Approach for Environmental Public Health Tracking

**Figure 1:**
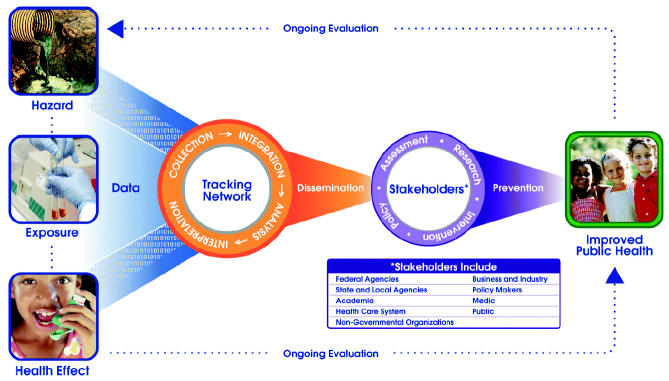
Environmental public health tracking. Reproduced from CDC (http://www.cdc.gov/nceh/tracking/diagram.pdf).

Environmental public health tracking is the ongoing collection, integration, analysis, and dissemination of data from environmental hazard monitoring, human exposure tracking, and health effect surveillance (Figure 1). Currently, the Centers for Disease Control and Prevention (CDC) is leading an initiative to build a national EPHT network that will meld data from these three components into a network of standardized electronic data systems and will provide valid scientific information on environmental exposures and adverse health conditions and the possible spatial and temporal relations between them.

CDC and our partners are applying the conceptual model first proposed by Thacker et al. (1996) to design the EPHT network. This model outlines the causal pathway starting with a hazardous agent present in the environment, followed by a population exposed to this agent and receiving a dose, and ending with a clinically apparent adverse health effect. Hazard, exposure, and health effects tracking represent data collection points along this continuum. Collecting, analyzing, and disseminating data from any one of these types of data systems or a combination of them provide important information for public health practice and comprise environmental public health surveillance activities. Development of a national EPHT network depends on the availability, quality, timeliness, compatibility, and utility of existing hazard, exposure, and health effect data. Both Thacker et al. (1996) and the Pew Commission (Environmental Health Tracking Project Team 2000) describe data that could potentially comprise part of a national EPHT network, such as vital statistics and the Aeromatic Information Retrieval System. Improvements to existing data systems, development of new systems, and integration of the data from these systems will be required to fully implement this network.

Hazards include chemical agents, physical agents, biomechanical stressors, and biologic toxins that can be found in our air, water, soil, food, and other environmental media. Often, data regarding these hazards are collected for regulatory purposes, and the characteristics of data collected are mandated by federal or state statutes. Thus, the types of data collected, the frequency of data collection, the location of data collection, and the collection methods may be optimal for enforcement activities but are less than ideal for public health surveillance use. For example, the U.S. Environmental Protection Agency’s (EPA) Safe Drinking Water Information System/federal version (SDWIS/FED) collects data from states on drinking water utilities’ noncompliance with federal drinking water standards (U.S. EPA 2004b). These data allow the U.S. EPA to track contaminant levels and to determine whether new regulations are needed to protect human health. However, the utility of SDWIS/FED for environmental public health surveillance is limited because actual monitoring data are available at the federal level only when results exceed the maximum contaminant levels, and the consistency of data elements over space and time can vary (Niskar 2003). At a minimum, hazard data included in the national EPHT network will need to be obtained through routine standardized data collecting and reporting and must have ongoing quality control, appropriate geographic coverage for the population at risk, and be available in a timely manner.

Exposure tracking is the monitoring of individuals, communities, or population groups for the presence of an environmental agent or its metabolite. Exposure data can include estimates derived from hazard data through sophisticated modeling. For example, the U.S. EPA and the National Oceanic and Atmospheric Administration are leading a project to model source emissions data, air monitoring data, and meteorologic data to forecast population exposure to ozone (U.S. EPA 2004c). Another example is the work conducted by the National Cancer Institute to estimate county-level exposures and thyroid doses received by American citizens from iodine-131 in atmospheric nuclear weapons testing fallout (National Cancer Institute 1997). Exposure data can also include direct measurements of individual exposure obtained from use of personal monitors such as passive air samplers and personal radiation dosimeters. However, neither of these types of exposure data is currently available for tracking exposures in an ongoing, systematic manner.

Exposure tracking of biomonitoring data represents the only method that actually measures the presence of hazardous agents in the human body. This type of exposure data provides information on the levels of chemicals or their metabolites in human biologic specimens such as blood or urine. Depending on the chemical agent, these measurements can serve as indicators of recent, long-past, or cumulative exposure to a hazard. For example, chemicals such as benzene are metabolized and excreted rapidly from the body, whereas the lipophilic compounds tetrachlorodibenzo-*p*-dioxin and polychlorinated biphenyl are retained for years (Thornton et al. 2002). Although more research is needed to understand fully the complex relationship between external hazard concentrations, internal dose, and health effects, biomonitoring data are an important component for comprehensive environmental public health surveillance.

Currently, few biomonitoring data are being tracked. At the national level, human samples are collected through the National Health and Nutrition Examination Survey and analyzed and reported by CDC in the “National Report on Human Exposures to Environmental Chemicals.” Blood and urine levels of 116 environmental chemicals are currently available for a sample of the noninstitutionalized U.S. population (CDC 2003). At the state level, state laboratories have limited capacity for biomonitoring. Childhood blood lead levels are the only measures that are routinely collected across most states, and these levels are collected in screening programs of high-risk children.

The final component in the conceptual model of Thacker et al. (1996) is health effects tracking, which represents traditional public health surveillance efforts. Disease registries, vital statistics data, annual health surveys such as the National Health Interview Survey, and administrative data systems such as hospital discharge data are sources that have been used for tracking health conditions. These varied sources have created a patchwork of health effect measures, and reliance on these data demonstrates the need for standardization for most disease surveillance. EPHT limits itself to those health effects with scientific evidence of possible environmental etiology. Health end points recommended as starting points for a national EPHT network by the Pew Commission focus on the following chronic conditions: birth defects; developmental disabilities such as cerebral palsy, autism, and mental retardation; asthma and other chronic respiratory diseases such as bronchitis and emphysema; cancer; and neurologic diseases, including Parkinson disease, multiple sclerosis, and Alzheimer disease. Additionally, the commission recommended tracking sentinels of exposures and health outcomes requiring rapid public health responses such as heavy metal poisoning and pesticide poisoning (Environmental Health Tracking Project Team 2000).

A key distinction between EPHT and traditional surveillance is the emphasis on data integration across health, human exposure, and hazard information systems (Figure 1). Our program to build a national EPHT network is the first national effort to provide the United States with standardized data from multiple health, exposure, and hazard information systems, that includes linkage of these data as part of regular surveillance activities. The network builds on separate ongoing efforts within the public health and environmental sectors to improve health surveillance, hazard monitoring, and response capacity (CDC 2004; U.S. EPA 2004a). This system will be used to identify potential relations between exposure and health conditions that either indicate the need for additional research or require intervention to prevent disease, disability, and injury.

### Network Vision and Strategy

The national EPHT network is still being formulated. However, expanding on the work of Hertz-Picciotto (1996), an ideal environmental public health surveillance system should include the following elements:

- Data systems that use compatible data standards and vocabularies
- High-quality, timely mortality and morbidity data with high resolution geographic coordinates
- A wide range of exposure information based on biomonitoring, personal monitoring, and exposure modeling
- Relevant, high-quality, and timely emissions data and monitoring data for air, water, soil, food, and other environmental media as well as geographic and temporal characteristics
- Access to population data, including information on migration and sociodemographic factors
- Tools to link data geographically
- Tools for descriptive and small-area analyses
- Tools for data dissemination
- Support for public health action

CDC and our partners are endeavoring to achieve the ideals listed above. We envision a tracking network that will be multi-tiered with functional components at the local, state, and federal levels. The main building blocks of the network will be statewide EPHT networks (or city-wide in the case of large municipalities) and national data surveys. As a major component of CDC’s Public Health Information Network (PHIN), the national EPHT network will be standards-based and compliant with the federal health architecture being developed by the Department of Health and Human Services (CDC 2004; Office of Management and Budget 2004). Additionally, it will be compatible with the U.S. EPA National Environmental Information Exchange Network (U.S. EPA 2004a) to facilitate bridging the current gap between health and environmental data.

As conceptualized, the network will include a core set of linkable health, exposure, and hazards data systems as well as data that have already been linked at local, state, regional, and national levels. CDC and our partners are currently evaluating the network’s priorities. At the federal level, implementation of the tracking network will require that CDC be able to access agreed-upon state and national data. Individually identifiable information will not be available at the federal level for surveillance purposes, and, at all levels, privacy will be protected. At the state and local levels, the network structure will be flexible enough to allow states to track their own unique priority issues as well as core national diseases, exposures, and hazards. The network will allow direct electronic data reporting and linkage within and across health effects, exposure, and hazard data while protecting confidentiality of individual records. Also, the network will enable exchange and aggregation of data across states.

The demand for better information about our environment and health comes from the public, the media, researchers, and policymakers. Although a main goal of the network is to make information available to a wide variety of stakeholders, state and federal privacy laws will restrict the types of information available to specific users.

### Building Bridges

**Figure 2:**
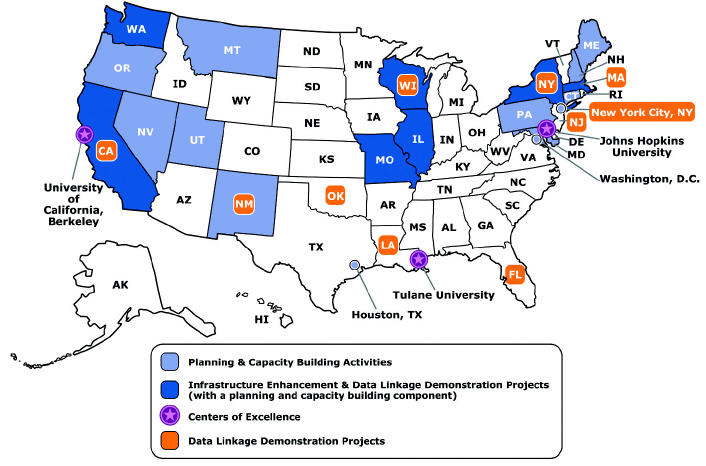
CDC’s EPHT Program grantees, 2004. Reproduced from CDC (http://www.cdc.gov/nceh/tracking/aag04.pdf).

Developing and maintaining partnerships are essential to building and sustaining the national EPHT network. Before the initiation of the tracking program, federal, state and local public health and environmental agencies, nongovernmental organizations, and academic institutions provided recommendations to CDC and the Agency for Toxic Substances and Disease Registry (ATSDR) that were incorporated into program development (CDC/ATSDR 2002). Collaborative activities continue to support the development of the national EPHT network as its infrastructure and methods are being developed and evaluated. Since 2002, CDC has funded 21 state health departments, three local health departments, and three schools of public health to conduct activities that will form the basis of a nationwide tracking network (Figure 2). The schools of public health are developing methods and conducting epidemiologic studies to advance the science of environmental public health that underlies the network and providing support to state and local partners. Eleven state partners and New York City are conducting projects to demonstrate *a*) an approach for linking existing health effect surveillance data with exposure or hazard data as part of ongoing surveillance activities, *b*) a sustainable effort to build capacity, and *c*) the usefulness of linked data in guiding public health policy and practice. Other state and local partners are conducting planning and capacity-building activities. In this mini-monograph, we present initial results from some of these projects.

Additionally, we are collaborating on improving communications and disseminating information about the national EPHT network with national professional organizations and advocacy groups, including the Association of State and Territorial Health Officials (ASTHO), the National Association of County and City Health Officials (NACCHO), the Environmental Council of States, the National Environmental Health Association, the Association of Public Health Laboratories, the Council of State and Territorial Epidemiologists, Physicians for Social Responsibility (PSR), and the Trust for America’s Health. For example, NACCHO is developing and circulating educational materials about EPHT to their constituency; ASTHO is serving as a conduit of information among CDC, state grantees, Centers of Excellence, and the unfunded states; and PSR is collaborating with NACCHO to increase the knowledge base and technical skills of physicians with regard to EPHT.

At the national level, both the U.S. EPA and the National Aeronautics and Space Administration (NASA) are active partners in development of the tracking network. As a cornerstone of this collaborative commitment, the U.S. EPA and CDC are taking advantage of the work being done on the U.S. EPA’s Exchange Network and CDC’s national EPHT network to increase health and environmental infrastructure and capacity at the local, state, and national level; to evaluate and improve data compatibility; and to collaborate on projects that develop and validate methods and tools to estimate exposure to environmental hazards for state and local partners. CDC and NASA also are working together to explore innovative public health applications of NASA technology such as existing remote sensing data collected via satellites for use in EPHT.

All three agencies (CDC, U.S. EPA, and NASA) are also collaborating in a new effort initiated by the CDC called Health and Environment Linked for Information Exchange, Atlanta (HELIX-Atlanta). We are coordinating this project with more than 70 representatives from local, state, federal, and academic partners. The public health significance of HELIX-Atlanta is to provide information regarding the five-county metropolitan Atlanta area through a network of integrated environmental monitoring and public health information systems so that all sectors can take action to prevent environmentally related health effects.

## Challenges and Expectations for Environmental Public HealthTracking

A neglected public health infrastructure and the lack of a trained workforce are monumental challenges to establishing a national EPHT network. In 1988 the Institute of Medicine (IOM) referred to the American public health system as a “shattered vision” (IOM 1988). Fifteen years later, a follow-up IOM committee found that improvements in the public health infrastructure and workforce development were still needed to ensure provision of essential public health services and to address emerging public health issues (IOM 2003). A trained, motivated, and dedicated workforce will be a necessity not only for the establishment of a national EPHT network but also for ensuring the health of the American people through the coming decades.

Depending on resource availability, the national EPHT network may need to build some surveillance infrastructure from the ground up. Surveillance systems currently do not exist at the local, state, or national levels to track many of the exposures and health effects that may be associated with environmental hazards (CDC 1998, 2000). Kass et al. (2004) describe the challenges of pulling together pesticide data for environmental public health surveillance. When information systems do exist, quality of the data, data vocabularies, and case definitions vary, and electronic reporting is still not an option for all sources of data (Goldman et al. 1992; Steenland and Savitz 1997). Laflamme and VanDerslice (2004) discuss the lack of standard environmental health questions in the Behavioral Risk Factor Surveillance System. Mather et al. (2004) present a table describing uses and limitations of hazard, exposure, and health effects data. These limitations present significant challenges to developing a national integrated data network. Standardization of technology and data specifications is ongoing within CDC (CDC 2004) and the national EPHT network is part of this effort. CDC and its partners are currently identifying data needs of their constituents and evaluating mechanisms and costs for improving data and filling data gaps.

The national EPHT network has received attention from the media, elected officials, and the public. Along with that attention has come some misunderstanding of the capacity of the network to provide answers to etiologic questions. As a surveillance system, EPHT cannot answer questions about causes of diseases. It can, however, generate hypotheses about etiology and identify areas where additional research is needed. We expect the national EPHT network will provide information to estimate the magnitude of a health effect in the population at risk, to detect epidemics or clusters, to document the distribution and spread of a health effect, to evaluate interventions, and to facilitate planning (Teutsch 2000).

Being able to link health, exposure, and hazard data on an ongoing basis will enable environmental public health practitioners to evaluate the spatial and temporal relations between environmental factors and health. However, detecting even these ecologic relations through the network will require careful analysis and interpretation. The pitfalls of drawing etiologic conclusions based on these ecologic relations are well documented and include issues such as confounding, measurement error, variation in event classification, and migration patterns (English 1996; Greenland 2004). In this mini-monograph, Mather et al. (2004) provide further discussion on ecologic bias in describing the statistical framework for analyzing the exposure, hazard, and disease relationship.

One factor to consider in interpreting relationships seen from analysis and visualization of tracking data is the lag time for most health effects thought to be associated with exposure to environmental hazards. Disease may occur in a specific area during a prescribed time, but exposure associated with the disease may have occurred months, years, or even decades earlier. Other factors contribute to the difficulties in characterizing environmental exposures because of the uncertainties inherent in exposure modeling and the mobility of the American public, whether related to daily commutes or relocating residence. Finally, many of the health effects of interest for environmental public health have multi-causal pathways. Other factors will confound or modify our ability to interpret the role of specific environmental exposures on disease risk when analyzing surveillance data.

### Lessons Learned in Building the EPHT Network

In this mini-monograph, we present the broad scope of activities of our partners to develop and evaluate methods for the science of EPHT. An initial step in building a national EPHT network has been to determine a core set of priority environmental public health problems and to identify existing data to describe these problems. State and local priority-setting activities, in combination with the recommendations from the Pew Environmental Health Commission (Environmental Health Tracking Project Team 2000), the Healthy People 2010 Objectives (U.S. Department of Health and Human Services 2000), and environmental public health indicator efforts (CSTE 2004; U.S. EPA 2003) will assist in identifying the core priority health, exposure and hazard data for the national EPHT network. Litt et al. (2004) describe the approach used by the Pew Environmental Health Commission as a model for priority setting, including the availability of existing data as a useful criterion for setting priorities.

State, local, and national data linkage demonstration projects are useful in identifying data gaps and compatibility issues in the environmental public health surveillance infrastructure. To fill data gaps, EPHT partners are collaborating to evaluate existing information systems that are not traditionally used for public health surveillance. Common challenges include data access, data completeness, data quality, and methods for integration that address temporal and spatial factors. This mini-monograph presents results of some unique and innovative methods for adapting and integrating non-traditional information systems for use in EPHT. For example, the work of Knorr et al. (2004) demonstrates the feasibility and utility of working with school nurses to get a more reliable estimate of asthma prevalence in school-age children at the local level than was previously available through traditional sources.

Data system compatibility issues are being addressed through ongoing work at the federal and state levels to standardize data vocabularies and messaging so that various data systems will “speak the same language.” CDC’s PHIN provides the architectural framework and specifications for the national EPHT network. The work of Hanrahan et al. (2004) on development of a PHIN-compliant module for Wisconsin’s childhood cancer surveillance project may serve as a model for other states and illustrates the challenges of designing an integrated data repository.

## Summary

EPHT is the ongoing collection, integration, analysis, and dissemination of environmental hazard, exposure, and health data. CDC and our partners are developing this approach for the EPHT network based on concepts from infectious and chronic disease surveillance and environmental hazard monitoring. The unique feature of the national EPHT network is the emphasis on data integration and standardization from all sources to improve data utility to the end user. With adequate funds, the EPHT network will provide valid scientific information on environmental exposures and adverse health effects that will bridge the existing data gap and provide a foundation for actions to improve community health. A key component of the EPHT network is dissemination. This mini-monograph is an opportunity to disseminate the first lessons learned about the innovative process of developing a national EPHT network. The articles represent the diversity of partners, priorities, and activities that are underway to build the EPHT network and to provide more information to the American people on how the environment contributes to human health.
